# Understanding the impact of introduction of *Robinia pseudoacacia* on community functional structure and moisture regulation in the Loess Plateau, China, using a trait-based approach

**DOI:** 10.3389/fpls.2024.1472439

**Published:** 2024-11-21

**Authors:** Cheng Zheng, Liuhuan Yuan, Haijing Shi, Gaohui Duan, Yangyang Liu, Zhongming Wen

**Affiliations:** ^1^ College of Grassland Agriculture, Northwest A&F University, Yangling, Shaanxi, China; ^2^ Institute of Soil and Water Conservation, Chinese Academy of Sciences and Ministry of Water Resources, Yangling, Shaanxi, China; ^3^ Institute of Soil and Water Conservation, Northwest A&F University, Yangling, Shaanxi, China

**Keywords:** response-effect trait framework, degraded ecosystem, moisture regulation, diversity, afforestation, forest management

## Abstract

Depending on specific environmental conditions, *Robinia pseudoacacia* plantations can have a positive or negative impact on ecosystem function. Numerous studies have demonstrated that *R. pseudoacacia* plantations on the Loess Plateau has decreased the water levels in this area, increasing the risks of water resource security. Understanding the ecosystem function of the *R. pseudoacacia* plantations is thought to be critical to vegetation restoration in the Loess Plateau. However, no consensus exists on the mechanism by which afforestation affects moisture regulation under varying environmental conditions nor on how to manage *R. pseudoacacia* plantations to maintain the ecosystem function. In this study, we used the response–effect trait approach to examine the evolving relationship between community functional composition and water regulation by collecting community samples from *R. pseudoacacia* plantations and natural ecosystems across three vegetation zones (steppe, forest–steppe, and forest). Our goal was to clarify how the afforestation of *R. pseudoacacia* impacts functional composition and, consequently, moisture regulation. The findings indicated that *R. pseudoacacia* negatively impacts community structure and moisture regulation in the drier steppe and forest-steppe (*P*<0.05). Afforestation of *R. pseudoacacia* increases specific leaf area (SLA), leaf nitrogen content (LNC), and plant height (H), while weakening the trait correlations within the community, which is the main cause of the negative effect. Furthermore, we discovered that response and effect traits overlapped (leaf tissue density, LTD) in natural ecosystems but not in afforested ecosystems within the response–effect traits framework. In conclusion, our findings indicated that the functional structure of communities and moisture regulation are impacted *R. pseudoacacia* plantations in drier habitats. Additionally, because response–effect traits do not overlap and trait coordination declines, afforestation increases instability in the moisture regulation maintenance. The introduction of *R. pseudoacacia* weakens the coordination and coupling relationships between traits. We advise giving preference to native species over *R. pseudoacacia* for restoration in the dry steppe and forest-steppe zones. Trait-based restoration approaches can enhance the efficacy of restoration measure in achieving desired ecosystem functions.

## 1 Introduction

Afforestation has emerged as a key nature-based solution for restoring degraded(1) ecosystems worldwide (Yu et al., 2019; Lu et al., 2020; Yan et al., 2023). Since 1999, China has completed 7.07 million hectares of afforestation, with the Loess Plateau accounting for 40% of the new green area (Han et al., 2020). This large-scale afforestation has significantly altered the material and energy balance of land surface, as evidenced by decreased erosion and sedimentation, increased vegetation coverage, and regional climate change (Liu et al., 2016; Wang et al., 2016; Fang et al., 2019). However, the rapid increase in the vegetation coverage on the Loess Plateau consumed substantial water resources, and water availability is approaching its upper limit (Feng et al., 2016; Jin et al., 2019). The excessive depletion of water resources from afforestation not only jeopardizes its sustainability (Vallejo et al., 2012; Lu et al., 2018; Liu et al., 2022) but also poses great risks to social and economic development (Cao et al., 2009). As a result, understanding community structure and moisture regulation has become critical in afforestation management and ecosystem function enhancement (Benayas et al., 2009).

*Robinia pseudoacacia* was introduced to the Loess Plateau in the 1950s and quickly became a pioneer tree species for vegetation restoration due to its rapid growth and high drought tolerance (Shangguan, 2007; Wang et al., 2020). *Robinia pseudoacacia* is widely planted as a plantation species around the world. Nevertheless, there is an ongoing debate concerning the ecological value of *R. pseudoacacia* plantations (Wu et al., 2015; Zhao et al., 2017; Ho et al., 2023). On the Loess Plateau, *R. pseudoacacia* plantations frequently exbibit degraded growth during the late-successional recovery stage, such as low biomass accumulation and small tree diameters. These plantations frequently become stunted and aged, producing trees with low ecological and economic benefits (Deng et al., 2016), thus failing to meet expected ecological functions. The primary reason for the low afforestation effectiveness and high water consumption is that afforestation often fails to consider site conditions (Tölgyesi et al., 2020). Mismatching the species with site may lead to significant soil drying due to its strong water absorption capacity (Liang et al., 2018; Yang et al., 2022). A thorough understanding of the structure and function of afforested ecosystems under various site conditions is critical for reversing the negative impact of *R. pseudoacacia* afforestation and improving afforestation management practices.

The introduction of *R. pseudoacacia* alters the original community structure and site conditions, influencing moisture regulation (Sitzia et al., 2012; Slabejova et al., 2019). The effects of *R. pseudoacacia* on plant diversity and composition have attracted significant interest from ecologists and conservationists (Sitzia et al., 2012; Piwczynski et al., 2016). However, species-based studies are often too slow to detect environmental changes and fail to provide real-time insights into afforestation’s impact on the ecosystem structure. In contrast to species composition, plant functional traits are more responsive to environmental changes (Koide et al., 2014; Swenson, 2016). Functional traits link individual plants to their environment (Chai et al., 2015; Funk et al., 2017). Correlations between plant functional traits have been used to identify functional constraints and trade-offs that underpin key plant ecological strategies in vegetation (Reich, 2014; Li et al., 2015). These functional traits are individual adaptations to change in in local or regional environmental gradients (Ackerly, 2004; Violle et al., 2007). Plant functional traits have been shown to be closely linked to soil moisture (Gross et al., 2008). To adapt to environmental spatial heterogeneity, individuals must adjust specific leaf area, leaf thickness, leaf dry matter content, and other traits in response to varying soil moisture conditions (Herberich et al., 2017; Li et al., 2021). Functional trait-based approaches, which focus on ecological processes and species’ quick responses to environmental changes, can transcend species classifications and thus the species status in restoration ecology (Laughlin, 2014; Rosenfield and Muller, 2017). In fact, there are still very few case studies that use functional traits to guide restoration efforts in restoration ecology.

**Figure 1:**
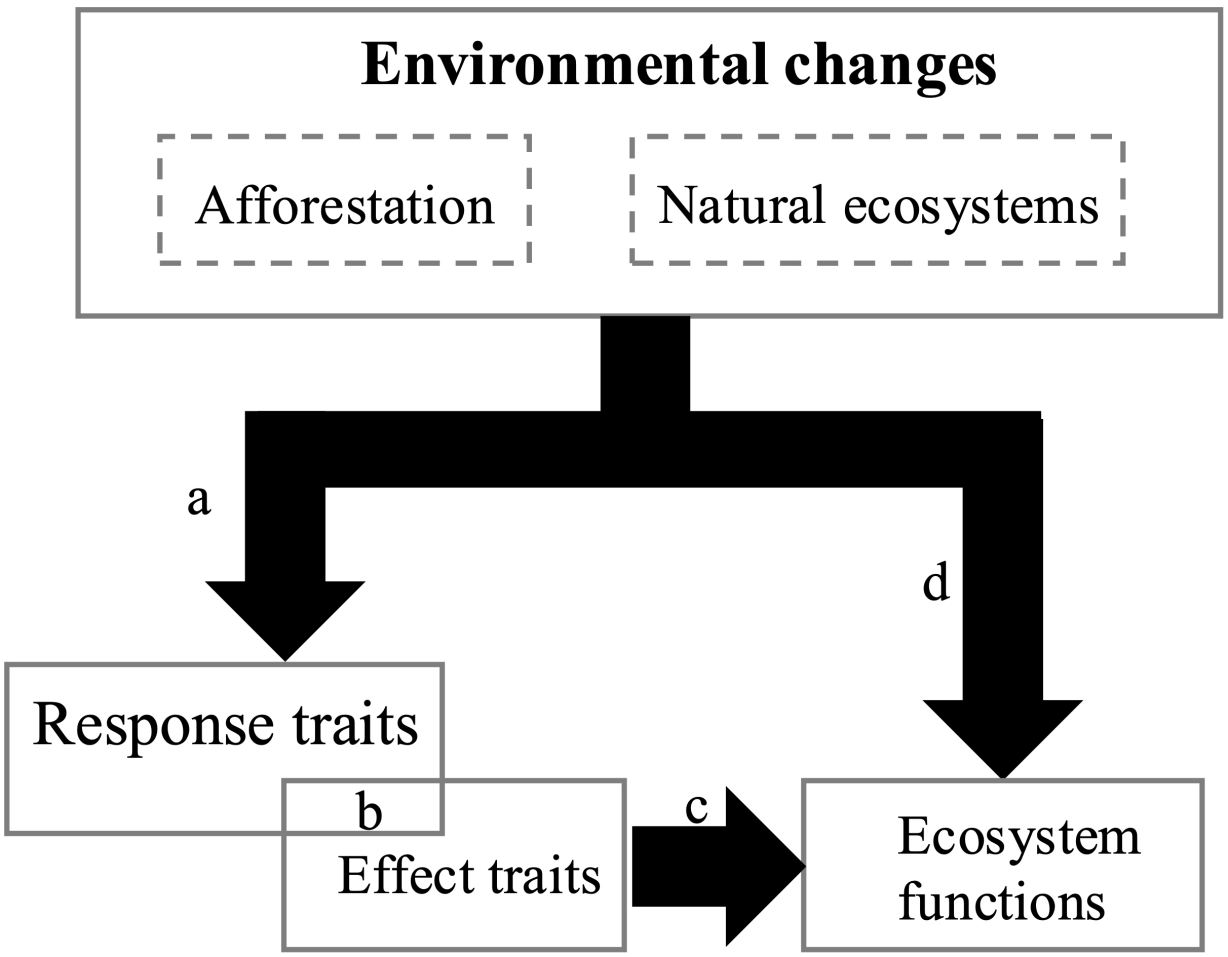
Conceptual framework of response effect traits: **(A)** environmental influence on traits. **(B)** Relationship between response traits and effect traits. **(C)** Effects traits on ecosystem function. **(D)** Independent effects of environment on ecosystem functions.

Functional traits are divided into response traits and effect traits (Violle et al., 2007). Response traits are biological characteristics related to environmental factors, such as disturbances and resources, whereas effect traits determine the impact of a species on one or more ecosystem functions (Lavorel and Garnier, 2002; Suding and Goldstein, 2008). The response–effect trait framework uses this trait classification to determine how environmental change affects plant community traits, which, in turn, may impact ecosystem functioning (Klumpp and Soussana, 2009; Solé-Senan et al., 2017; Hu et al., 2021).The response–effect trait framework is used to quantify species interactions and ecosystem functions (Lindo, 2015; Refsland and Fraterrigo, 2017). The relationships between response and effect traits may overlap, be correlated, or be independent, depending on the context, traits, and ecosystem functions selected (Suding et al., 2008; Zirbel et al., 2017). Trait associations at the community level reveal community assembly processes, which are important for understanding community structure and functional processes (Silvertown, 2004; Kooyman et al., 2010; Gotzenberger et al., 2012). For instance, Lasky et al. (2014) found that stable niche differences related to specific leaf area and leaf dry matter content mediate competition. As a result, we present a theoretical framework based on response–effect traits, which includes both direct effects of environmental changes and the indirect effects mediated by functional traits on ecosystem functions (**Figure 1**). By comparing the response–effect model of *R. pseudoacacia* plantations and natural ecosystems along the same vegetation gradient, we explored the mechanisms by which *R. pseudoacacia* plantations affect moisture regulation and provided recommendations for vegetation restoration practices.

Water resource security is a primary concern in arid and semi-arid regions worldwide (Jia et al., 2017; Zhang et al., 2021). Issues caused by afforestation on the Loess Plateau have drawn widespread attention (Deng et al., 2016; Liu et al., 2018; Han et al., 2020). However, while macro-environmental changes affect water resource distribution, it remains uncertain how changes in community structure due to afforestation influence water resource distribution. There is no consensus on how to manage existing plantations to ensure long-term moisture regulation. Using the response–effect trait framework, this study compared *R. pseudoacacia* plantations and natural ecosystems (steppe, forest-steppe, and forest according to environmental gradients) in the Yanhe River Basin (YRB) of the Loess Plateau. We investigated soil moisture and community samples and relationship between the community structure and soil moisture of the *R. pseudoacacia* plantations and natural ecosystems, aiming to clarify three key issues: (1) to investigate the impact of the introduction of *R. pseudoacacia* on the vegetation community structure and moisture regulation under different vegetation zones, (2) to reveal the mechanisms by which afforestation affects moisture regulation by comparing trait associations, and (3) to use the response–effect trait framework to compare ecosystem function maintenance processes in *R. pseudoacacia* plantations and natural ecosystems.

## 2 Materials and methods

### 2.1 Study area and sampling sites

**Figure 2:**
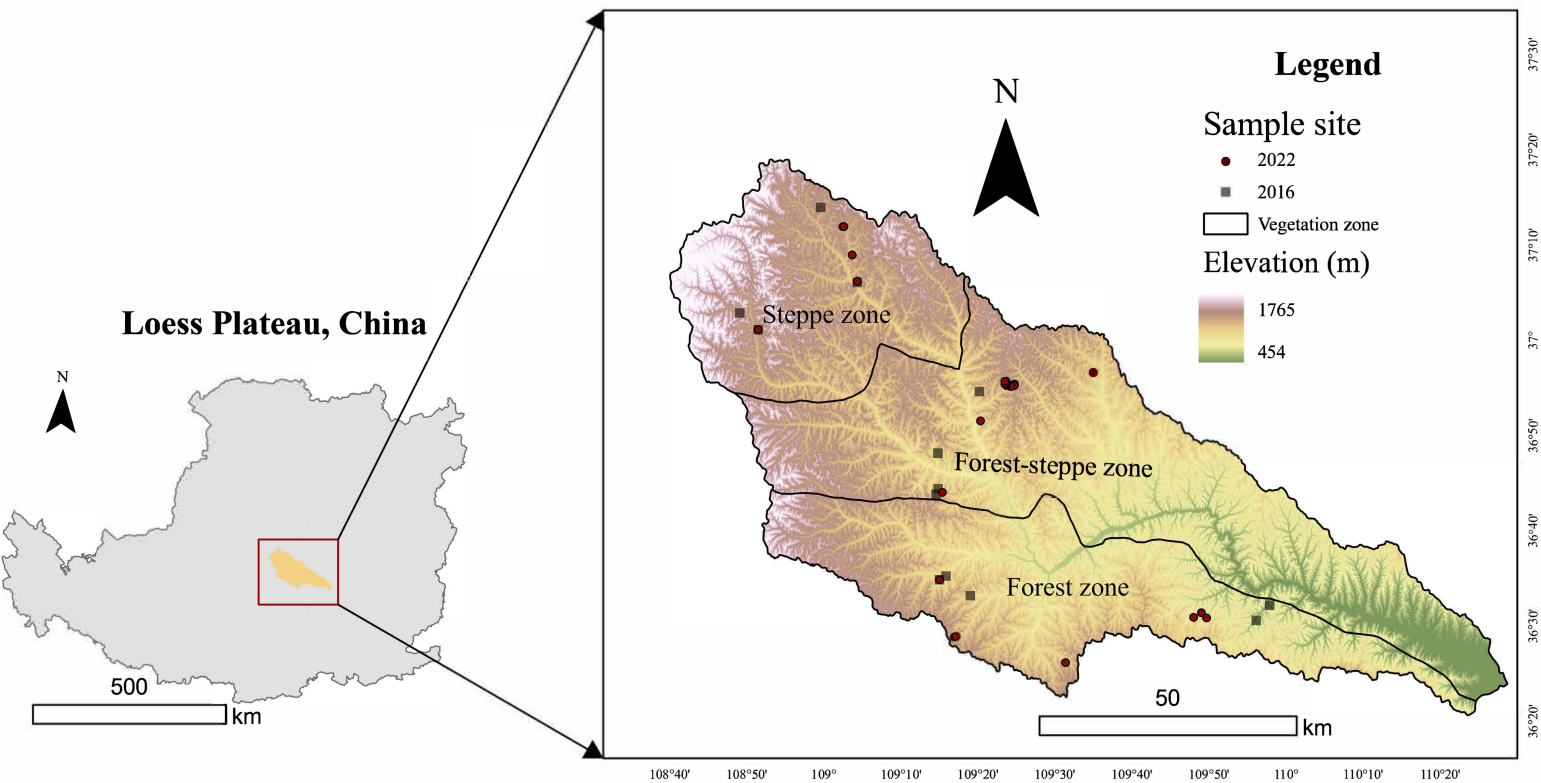
The spatial distribution map of the study sampling sites.

The study was conducted across the loess hilly-gullied landscape of Yanhe River Basin (YRB), which is located in the middle area of the Loess Plateau of China (latitude, 36°21′–37°19′N; longitude, 108°38′–110°29′E). It covers an area of 7,725 km^2^ (**Figure 2**), with annual temperature ranging from 8.8°C to 10.2°C and annual precipitation ranging from 450 mm to 500 mm over the last decade. The spatial and temporal variation of precipitation and temperature in YRB is obvious, which affects regional vegetation distribution. The dominant vegetation types are forest, forest–steppe, and steppe. The forest zone is dominated by *Quercus mongolica*, the forest–steppe zone by *Periloca sepium* and *Buddleja alternifolia*, and the steppe zone by *Stipa bungeana*, *Bothriochloa ischaemum*, *Poa sphondylodes*, and *Cleistogenes caespitosa*. YRB is one of the areas with the highest rate of soil and water loss on the Loess Plateau, with the soil and water loss area accounting for 80% of the basin area. The most commonly planted tree is *R. pseudoacacia*.

We selected 40 sites within the basin that had both natural ecosystems and *R. pseudoacacia* plantations in close proximity to ensure that biotic and abiotic conditions are comparable. We make sure that these natural ecosystems are far away from roads and settlements, minimizing human disturbance as much as possible. *Robinia pseudoacacia* plantations were planted more than 20 years ago and are adjacent to the natural ecosystem. Of these sites, 13 were from the 2016 field survey and 27 were from the 2022 survey. **Figure 2** shows the distribution of the study sites.

### 2.2 Sampling design

For each site, we created two 10 m × 10 m plots for investigation and sampling: one for *R. pseudoacacia* plantations and the other for natural vegetation. For sampling, three 1 m ×1 m subplots were used for the herbaceous layers (arranged diagonally in the large plot) and a 5 m×5 m subplot for shrubs (positioned in the middle of the large plot).

We investigated the height, abundance, coverage, and biomass of each species in each subplot. A total of 10 leaves of each species were collected and packed into plastic bags at the plot. They were then brought to the laboratory in an icebox to be measured for the leaf area and thickness. We collected at least 20 g of leaves from the plants and put them into plastic bags. All the samples were dried at 65°C for 48 h until they reached a constant weight, after which they were crushed. Finally, the carbon, nitrogen, and phosphorus content of the leaves was measured.

To measure soil moisture, we used the S-type route to select 5 points in the plot and collected the soil samples with a soil drill at three depths (0–10 cm, 10–20 cm, and 20–40 cm). The soil samples from the same depth were then mixed, placed in plastic bags, and brought back to the laboratory.

### 2.3 Functional traits and functional diversity

To estimate the functional structure of plant communities, we focused on six growth-related functional traits: plant height (H, cm), which is associated with a plant’s ability to compete for light, and specific leaf area (SLA, mm^2^/g), leaf tissue density (LTD, mg/mm^3^), leaf nitrogen content (LNC), leaf carbon content (LCC), and leaf phosphorus content (LPC), which indicate a species’ resource-use strategy. Plant functional trait data were collected from field sampling to avoid potential confounding effects arising from within-species trait variation. Species were derived from plot-scale species, which included nearly all local dominant species and rare species.

We calculated community-weighted means [CWMs, Garnier et al. (2004)], which are the average trait values of plant communities, and reflect the importance of species. The formulas for calculating CWMs are as follows:


(1) $$\begin{array}{c} P_{i}=\frac{Ra+Rb+Rc}{3} \end{array}$$

(2) $$\begin{array}{c} CWM_{ij}=\sum_{i=1}^{n}p_{ij}\times trait_{ij} \end{array}$$

where p_ij_ is the importance value of species i in plot j, Ra is the relative abundance, R_b_ is the relative biomass, and R_c_ is the relative coverage in plot j (Zheng et al., 2010). Trait_ij_ is the mean trait value of species i in plot j and n is the number of species in the plot. Functional diversity was assessed using the quadratic entropy index and functional diversity (FRic), which quantifies the species dissimilarity based on functional trait values (Luo et al., 2019). FRic of different traits was calculated by the functional range index proposed by Mason et al. (2005). Its calculation formula is:


(3) $$\begin{array}{c} FRic=\frac{SFci}{\text{Rc}} \end{array}$$

SF_ci_ is the niche space filled by the species within the community; R_c_ is the absolute range of the trait.

The Rao Quadratic Entropy (RaoQ) index is calculated using a distance matrix of functional traits and the relative importance value of species, where d*ij* represents the difference in functional traits between species i and j, and Pi and Pj represent the relative abundance of species i and j, respectively.


(4) $$\begin{array}{c} RaoQ=\sum_{i=1}^{S}\sum_{j=1}^{S}d_{ij}p_{i}p_{j} \end{array}$$

### 2.4 Statistical analysis

To assess the impact of introduction of *R. pseudoacacia* on plant community structure and soil moisture, we compared the soil moisture, functional traits, and diversity between *R. pseudoacacia* plantations and natural ecosystems, within each vegetation zone separately, using *t*-test. To assess the effect of major environmental gradients, we compared the soil moisture, functional traits, and diversity among the three vegetation zones (steppe vs. forest–steppe vs. forest), for both the *R. pseudoacacia* plantations and natural ecosystems separately, using homogeneity test of variance and ANOVA. The Pearson correlation coefficient was used to analyze trait association. The response–effects framework is built using mixed-effects models that look at both response and effect traits. To detect response traits, the change of vegetation zone was used as a fixed effect, and different years were used as a random effect to explain the non-independence of time effects, and various traits were tested. We estimated the parameters using restricted maximum likelihood estimation to, and we tested the model’s significance with the chi-square test. In the effect trait test, we used the MuMIn package to build a complete model including all of the traits tested. The lme4 package was used to complete the mixed effect model, and the optimal model is chosen using the Akaike Information Criterion (AIC) (Zuur et al., 2010). All statistical analyses were conducted using R.

## 3 Results

### 3.1 Functional composition and soil moisture distribution

**Figure 3:**
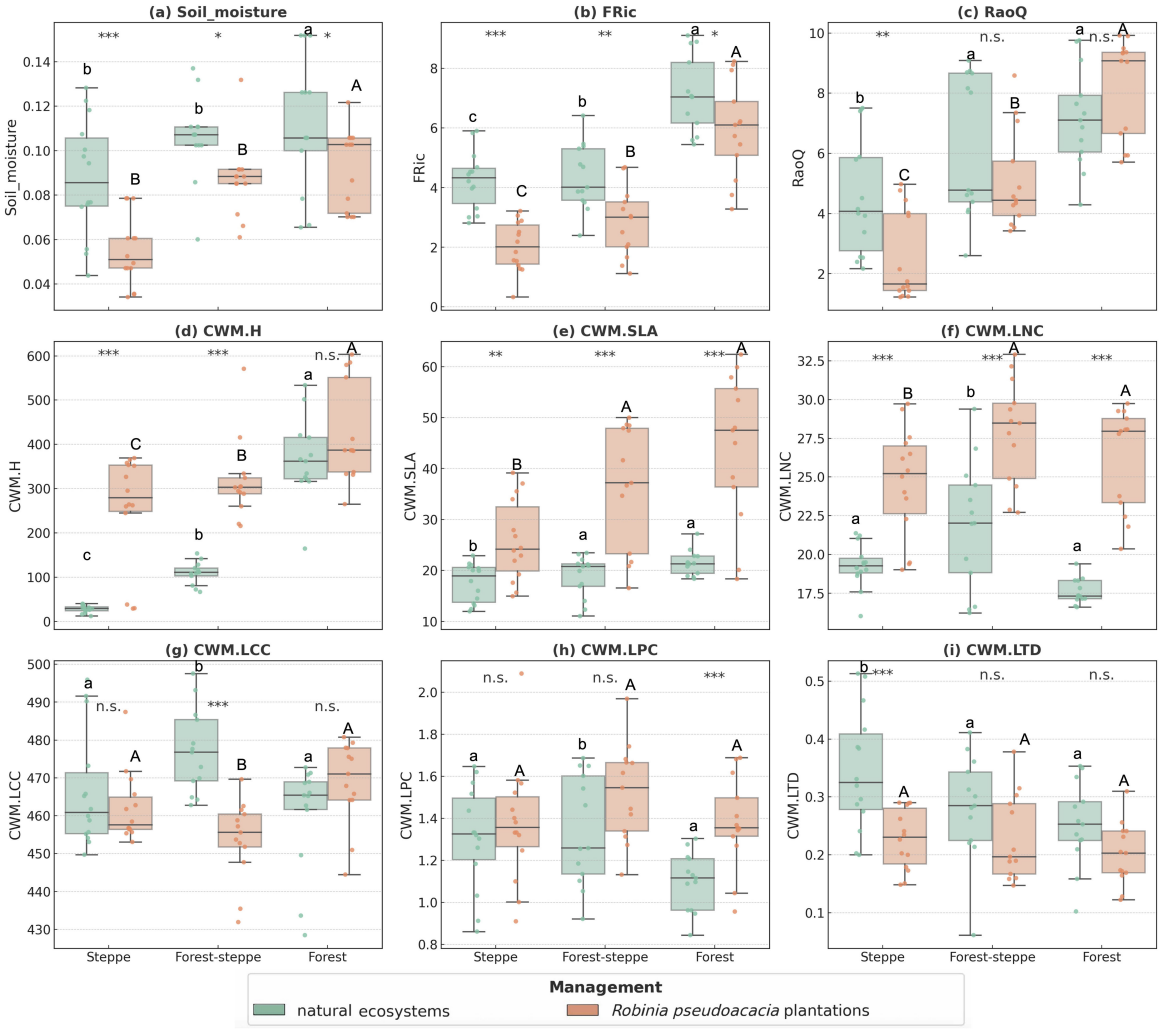
Functional composition indices and soil moisture distribution of *Robinia pseudoacacia* plantations and natural ecosystems. *, **, and *** indicate that the difference are significant at *p* < 0.05, *p* < 0.01 and *p* < 0.001. The same below. Lowercase and capital letters denote the variations among the vegetation zones for both natural ecosystems and plantations, separately. Panels **(A)** Soil moisture, **(B)** FRic, **(C)** RaoQ, **(D)** CWM.H, **(E)** CWM.SLA, **(F)** CWM.LNC, **(G)** CWM.LCC, **(H)** CWM.LPC and **(I)** CWM.LTD.

In comparison to natural ecosystems, *R. pseudoacacia* plantations had significantly lower soil moisture in all vegetation zones (*p*<0.05) (**Figure 3A**). In terms of functional diversity, we found that FRic of natural ecosystems are higher than that of *R. pseudoacacia* plantations in all zones, and RaoQ of natural ecosystems are higher in the steppe. Moreover, CWM.SLA and CWM.LNC are smaller in natural ecosystems than in *R. pseudoacacia* plantations in all zones; CWM.LCC is higher in natural ecosystems than in *R. pseudoacacia* plantations in the forest–steppe, CWM.LPC is smaller in natural ecosystems than in *R. pseudoacacia* plantations in the forest, and CWM.LTD is bigger in natural ecosystems than in *R. pseudoacacia* plantations in the steppe.

### 3.2 Trait association

**Figure 4:**
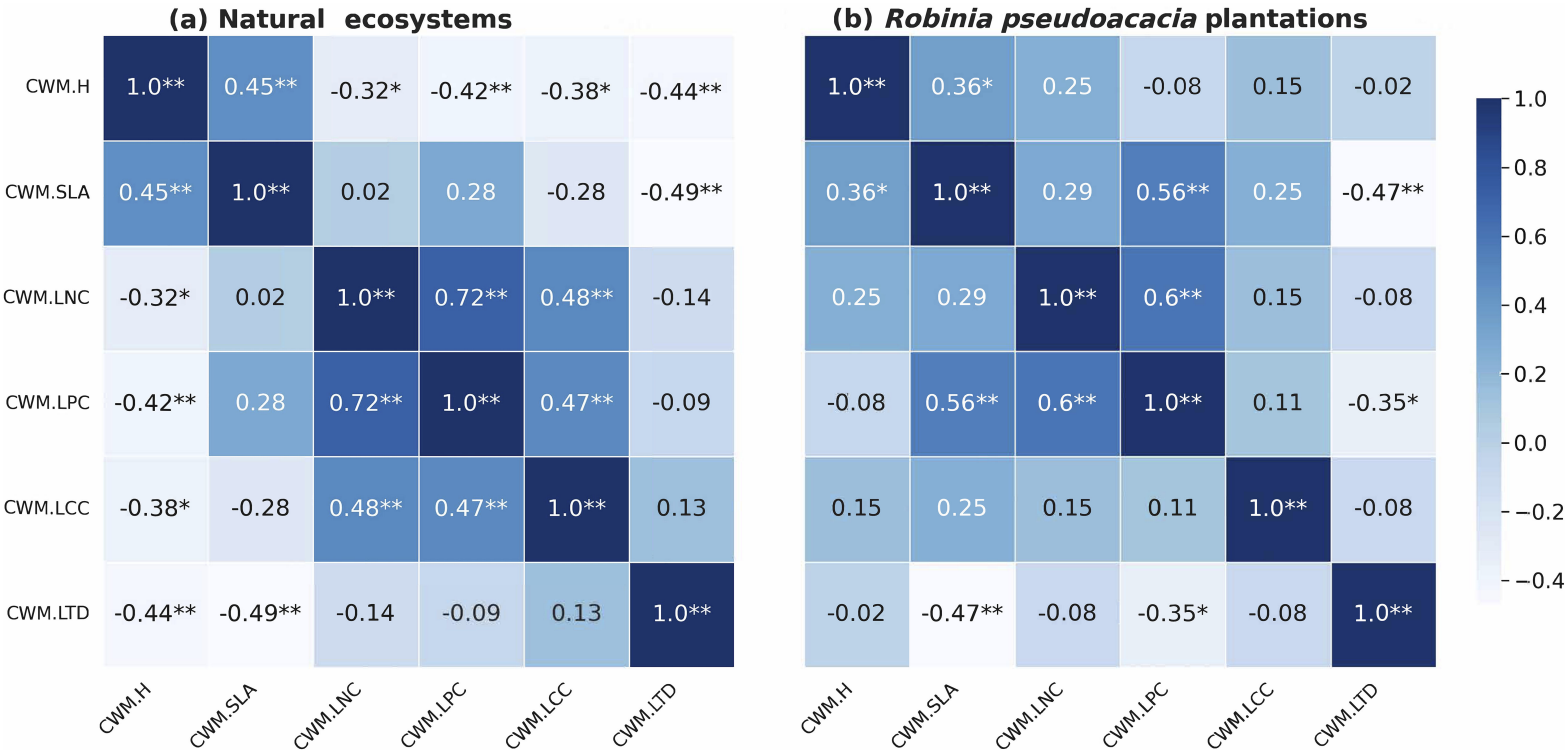
Correlogram of CWM metrics Pearson correlation. Correlation coefficients are shown in color and significance is marked. * and ** indicate the difference are significant at *P* < 0.05 and *P* < 0.01. Panels **(A, B)** represent the natural ecosystems and the *Robinia pseudoacacia* plantations, respectively.

We found varying degrees of correlation between CWM trait values across our ecosystems (**Figure 4**). In *R. pseudoacacia* plantations, CWM.LPC was negatively correlated with CWM.H and CWM.LTD, positively correlated with CWM.SLA and CWM.LNC, and CWM.LTD was negatively correlated with CWM.SLA, while CWM.H and CWM.SLA were positively correlated. In natural ecosystems, there is also a close correlation between leaf stoichiometry (CWM.LCC is positively correlated with CWM.LNC and CWM.LPC) and plant height (CWM.H was positively correlated with CWM.LNC and CWM.LPC) (*P*<0.05).

### 3.3 Responses of soil moisture and functional indicators to vegetation zones

Vegetation zones influence community functional structure of natural ecosystems (from steppe to forest). We found that soil moisture content and functional diversity metrics were lower in the steppe zone than in the forest zone (**Figure 3**). The mixed effects model shows that vegetation zone gradient was positively correlated with FRic and RaoQ, while CWM.H and CWM.SLA are negatively correlated with CWM.LTD and CWM.LPC. Other traits were not significant in the full model (*p* < 0.05) (**Table 1**).

The relationship between functional traits of *R. pseudoacacia* plantations along the vegetation zones is similar to that of natural ecosystems, with the exception of CWM.LTD and CWM.LPC, which were not significant in the full model test (*p* < 0.05) (**Table 1**).

**Table 1:** Linear mixed effect models testing the response of functional traits and functional diversity of (a) natural ecosystems and (b) *Robinia pseudoacacia* plantations to vegetation zones.

Response variable	RaoQ	FRic	CWM.H	CWM.SLA	CWM.LTD	CWM.LCC	CWM.LNC	CWM.LPC
(a) Natural ecosystems	**1.28(<0.01)**	**1.43(<0.01)**	**167.79(<0.01)**	**1.81(<0.01)**	**−0.04 (0.01)**	−2.98(0.30)	−0.81(0.16)	**−0.11 (<0.01)**
(b) *Robinia pseudoacacia* plantations	**2.82(<0.01)**	**1.96(<0.01)**	**87.08(<0.01)**	**8.72(<0.01)**	−0.01(0.29)	3.29(0.15)	0.82(0.24)	−0.01(0.90)

Estimates of coefficients, their 95% intervals (in parentheses), with bold indicating statistical significance (*p* < 0.05).

### 3.4 Effect of functional diversity and functional traits to soil moisture

A full mixed-effects model that integrated functional diversity and functional traits revealed that higher CWM.LTD was associated with lower soil moisture in natural ecosystems. Functional diversity (FRic) had a positive effect on soil moisture in the *R. pseudoacacia* plantations, while CWM.LNC had a negative effect (*p* < 0.05) (**Table 2**).

**Table 2:** Linear mixed effect models testing the response of functional diversity and functional traits of (a) natural ecosystems and (b) *Robinia pseudoacacia* plantations to soil moisture (log-transformed).

Response variable	RaoQ	Fric	CWM.H	CWM.SLA	CWM.LTD	CWM.LCC	CWM.LNC	CWM.LPC
natural ecosystems	0.003 (0.17)	0.005 (0.15)	−0.0001(0.03)	−0.004 (0.10)	**−0.17 (<0.01)**	−0.0005(0.27)	−0.0003(0.92)	−0.06 (0.18)
*Robinia pseudoacacia* plantations	1.580e−03(0. 30)	**6.168e−03(<0.01)**	−2.867e−05 (0.29)	5.615e−04 (0.20)	6.461e−02 (0.18)	**−1.085e−03 (<0.01)**	1.781e−03 (0.09)	−2.452e−02 (0.18)

Estimates of coefficients, their 95% intervals (in parentheses), with bold indicating statistical significance (p < 0.05).

## 4 Discussion

Understanding how community functional structure determines ecosystem function is a major goal of restoration ecology (Yang et al., 2019). Manipulating community structure to achieve functional goals is a key aspect of restoring degraded ecosystem (Laughlin, 2014). We present, to our knowledge, the first response–effect trait framework that integrates *R. pseudoacacia* plantations and natural ecosystems. Using quantitative trait-based approaches to explain community structure and ecosystem function yields more generalizable predictable outcome (Cadotte, 2017). First, we used natural ecosystems as a reference to investigate the effects of afforestation on community structure and moisture regulation in different vegetation zones. Afforestation alters the relationship between community structure and soil moisture, indicating that it has an impact on ecosystem. Second, we detected differences in trait correlations between natural ecosystems and *R. pseudoacacia* plantations, indicating that afforestation affects the community assembly and, consequently, ecosystem function. This finding provides important implications for understanding the relationship between community structure and ecosystem function. Finally, we used a mixed effects model to construct a response–effect trait framework for both ecosystems, which can support ecosystem management and restoration efforts, ultimately resulting in the desired ecosystem functions.

### 4.1 Effects of afforestation on community structure and soil moisture

Soil moisture varied significantly between vegetation zones. In most cases, we found the highest soil moisture in the forest zone and the lowest soil moisture in the steppe zone, indicating the spatial heterogeneity of regional precipitation (Yuan et al., 2023). Additionally, soil moisture in *R. pseudoacacia* plantations was significantly lower than in natural ecosystems across three vegetation zones, which is consistent with reports by Deng et al. (2016) and Dang et al. (2022). Afforestation is likely to coincide with the desiccation of deeper soil layers, leading to insufficient ground water recharge (Tölgyesi et al., 2020). One explanation is that *R. pseudoacacia* has larger roots and absorb more water for growth (Perez-Harguindeguy et al., 2013). Another explanation is that *R. pseudoacacia* has a larger canopy and broader leaves than native species, trapping rainfall and increasing evapotranspiration (Yang et al., 2014). We found that the overconsumption of water due to afforestation was more pronounced in arid steppe zone than in forest zone.

*Robinia pseudoacacia* plantations reduced functional diversity (FRic) in the relatively arid steppe and forest–steppe zones. *Robinia pseudoacacia* is an alien species that competes for resources and absorbs more water than native species (Weiher et al., 1999; Su and Shangguan, 2019). Ho et al. (2023) found that the *R. pseudoacacia* plantations had lower Shannon diversity and functional diversity than near-natural forests. The introduction of *R. pseudoacacia* altered resource allocation within the community, leading to changes in its functional structure (Cierjacks et al., 2013). In the arid loess hilly areas, water resource competition largely determines community composition. On the other hand, the introduction of *R. pseudoacacia* alters both biological and abiotic habitat environment, affecting factors such as soil microbial communities and microclimates (Zhou et al., 2022; Zhang et al., 2023). The functional diversity in afforested ecosystems is lower than in natural ecosystems, indicating that the *R. pseudoacacia* exerts a strong filtering effect on community structure (Pysek et al., 2012). In the forest zones, however, the impact of *R. pseudoacacia* plantations on functional diversity is comparable to that of natural ecosystems. This is consistent with the findings of Hu et al. (2021), indicating that *R. pseudoacacia* is expected to have less negative effect in environments with better hydrothermal conditions. To avoid depletion of water resource, we advocate for reducing the area of *R. pseudoacacia* plantations in arid areas.

The effects of afforestation on plant community structure and moisture regulation are closely related to plant functional traits (Qin et al., 2016). Non-native species have functional traits that differ from native species (Dyderski and Jagodzinski, 2019). Our findings showed that *R. pseudoacacia* plantations had higher CWM.SLA, CWM.H, CWM.LNC, and CWM.LPC than natural ecosystems. The values of these functional traits indicate how adaptable a species is to its surroundings (Cornelissen et al., 2003). Significant differences in functional traits were also noted across different vegetation zones, indicating that plant communities in these zones use distinct functional trait strategies to adapt to environmental variation (Luo et al., 2016; Ahrens et al., 2020). In comparison to forest zone, *R. pseudoacacia* plantations usually had a lower CWM.SLA in the arid steppe zone. Our hypothesis was that by affecting the community resource allocation in the steppe zone, *R. pseudoacacia* plantations may shifted the investment strategy of leaves from conservative to economical one. In addition, the environment conditions of different vegetation zones determine how *R. pseudoacacia* plantations impact the structure of plant communities. In the arid steppe zone with poor habitat conditions, *R. pseudoacacia* plantations had the most significant effect on soil moisture and functional diversity. In contrast, in the forest zone, where water conditions were favorable, the effects of *R. pseudoacacia* plantations were weak or even negligible. This suggests that *R. pseudoacacia’s* strong water-competitive ability under poor water conditions likely suppresses the native plant growth and interferes with the functioning of ecosystem. However, in the more resource-rich forest zone, the water scarcity is reduced, reducing *R. pseudoacacia*’s competitive advantage. In these conditions, *R. pseudoacacia* may even promote nutrient cycling and enhance ecosystem functions. This study highlights the risks of afforestation without considering habitat conditions and the traits of introduced species. It is essential to assess the ecological impacts of introduced species under changing environmental conditions to develop effective afforestation management strategies that enhance ecosystem service value.

### 4.2 Effect of afforestation on trait associations

Plant functional strategies are typically achieved through the simultaneous expression of various traits, and their associations are likely shaped by environmental filtering (Candeias and Fraterrigo, 2020). The coordinated expression of functional traits has an adaptive value, particularly when it comes to optimizing traits to water conservation. Numerous investigations have demonstrated that resource-limited environments restrict the variation and distribution of plant functional traits (Laliberté et al., 2014). Plants often adopt traits combinations to cope with environmental stresses (He et al., 2020). In arid hilly areas, stable correlations between sets of traits are common (Yin et al., 2018). In the natural ecosystems of the loess hilly region, the content of nitrogen and phosphorus in leaves changes closely and synergistically, which is consistent with the prediction of plant ecological stoichiometric theory: to achieve functional balance during plant growth, nitrogen and phosphorus must be coupled to form a stable nitrogen and phosphorus ratio (Gusewell, 2004). In addition, Burns and Beaumont (2009) discovered a coupling relationship between the maximum plant height and the leaf economic traits. The reason may be that the complex terrain in hilly areas affects the distribution of water and heat factors, which in turn affects plant photosynthesis and drought resistance adaptation mechanisms. However, the relationships among functional traits in *R. pseudoacacia* plantations were significantly weaker than those in natural ecosystems. The reason is that *R. pseudoacacia*, a nitrogen-fixing plant, disrupts ecosystem’s nutrient cycle, weakening the coordination between leaf chemical traits, especially the decoupling leaf nitrogen content with phosphorus content in *R. pseudoacacia* plantations.

### 4.3 Response–effect trait framework

In this study, we employed a response–effect trait framework to identify which combinations of functional trait structures achieve desired ecological benefits in *R. pseudoacacia* plantations ecosystems and natural ecosystems. In natural ecosystems, most leaf morphological traits responded significantly to environmental changes, as previously reported (Guo et al., 2021; Zheng et al., 2024). This is not surprising given that environment filtering is associated with functional traits (Lebrija-Trejos et al., 2010). Leaf functional traits either increased or decreased along the vegetation zone gradient. The introduction of *R. pseudoacacia* altered the relationship between some traits and the vegetation gradient. For instance, the significant relationships involving CWM.LTD and CWM.LPC disappeared. Regarding the effect traits, only CWM.LTD was significantly associated with soil moisture in the natural ecosystems, while no significant association was found between any functional trait and soil moisture in the *R. pseudoacacia* plantations.

**Figure 5:**
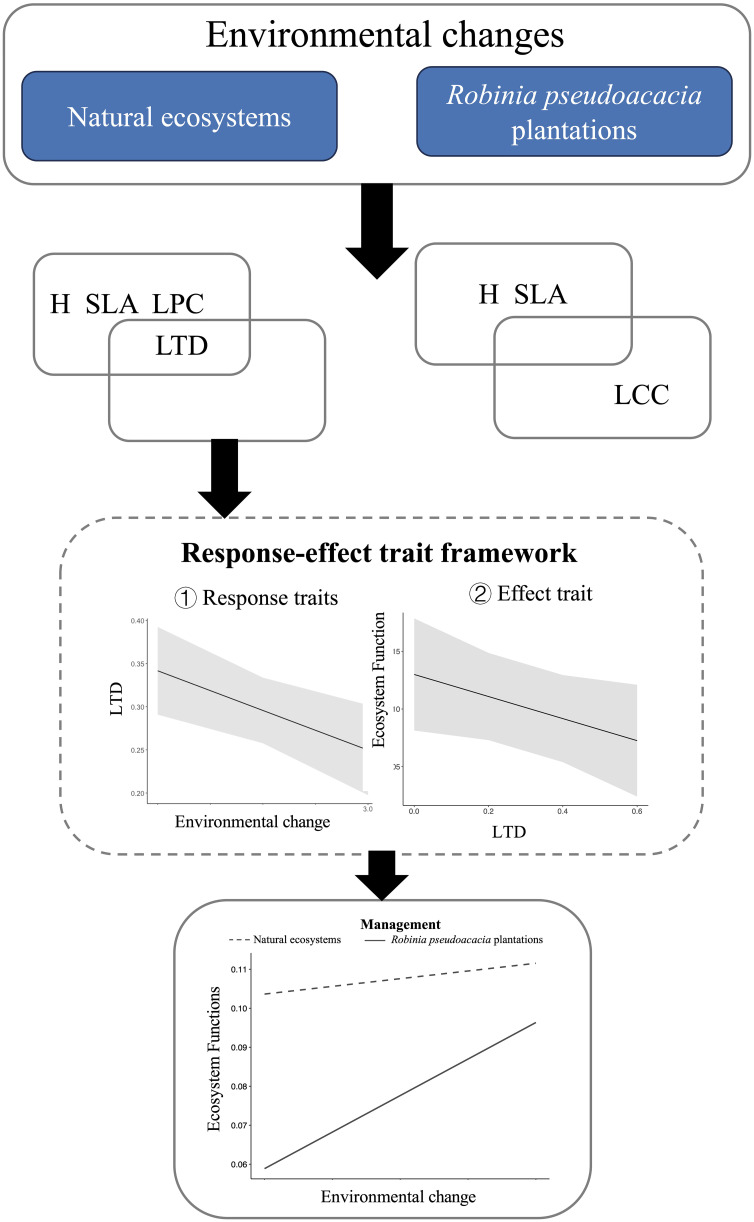
Summary of the effects of afforestation on changes in water resource availability, based on a response effect trait framework.

In this study, the response–effect trait framework for natural ecosystems revealed that CWM.LTD is a functional trait in which response traits and effect traits overlap. To avoid excessive consumption of water resources in natural ecosystems, a combination of species with CWM.LTD as small as possible should be selected. Furthermore, species with small LTD may be able to adapt to environmental changes. Nevertheless, afforestation is more likely to increase water resource consumption and impact moisture regulation in the drier steppe and forest–steppe zones of the YRB (**Figure 5**). In *R. pseudoacacia* plantations, we did not find any trait that would be a response trait and an effect trait simultaneously. Currently, the response–effect trait framework in *R. pseudoacacia* plantations is more about the diversity of multiple functional traits rather than single functional traits. This framework proposed a management strategy for the existing problem of afforestation ecosystems in the Loess Plateau, namely, selecting specific components with high functional diversity to reduce the excessive consumption of water resources or reducing the planting area of *R. pseudoacacia* plantations.

The correlation between response traits and effect traits is crucial for understanding how community assembly processes indirectly affect ecosystem functions (Zirbel et al., 2017). When response and effect traits are identical or related, predictability can be established based on the relationship between trait set and functions (Lavorel and Garnier, 2002). In degraded ecosystems, restoration practitioners can use management techniques to modify specific environmental conditions to achieve targeted ecosystem services (Fu et al., 2023). However, response and effect traits may not be correlated, limiting the ability to predict ecosystem functions. For example, in *R. pseudoacacia* plantations, we found no effect traits associated with response traits. In such cases, predicting moisture regulation based on commonly measured functional traits or trait response mechanisms may not be possible. Future work should consider additional traits that may influence community assembly and ecosystem functions, for example, root traits and taxonomic traits. *Robinia pseudoacacia* is not the only tree species used for revegetation in YRB. One of the urgent tasks in ecology research is comparing the ecological value of afforestation with different species.

## 5 Conclusions

The introduction of *R. pseudoacacia* has a significant influence on the functional structure and moisture regulation of the dry steppe and forest–steppe zones. Our results showed that *R. pseudoacacia* plantations affected the distribution of traits and weakened the correlation between traits compared to the natural ecosystems, affecting community structure and moisture regulation. In addition, we propose a practical transformation scheme for forest management to achieve ecosystem functional expectations by introducing a trait response–effect framework for both natural ecosystems and *R. pseudoacacia* plantations, despite the fact that our findings did not find response–effect traits in *R. pseudoacacia* plantations. The afforestation area of *R. pseudoacacia* plantations in the steppe and the forest–steppe zones must be reduced if soil moisture balance is to be maintained during ecological restoration.

## Data Availability

The original contributions presented in the study are included in the article/supplementary material, further inquiries can be directed to the corresponding authors.
